# Preparation and application of melamine cross-linked poly ammonium as shale inhibitor

**DOI:** 10.1186/s13065-018-0410-9

**Published:** 2018-04-25

**Authors:** Li Zhang, Tiehu Li, Lei Huang, Zhengqin Ye, Zhongbin Ye, Xin Yan, Lili Li, Qiang Deng, Gang Chen, Jie Zhang, Zhifang Zhang

**Affiliations:** 1 School of Materials Science and Engineering, Northwestern Polytechnical University, Xi’an, 710072 China; 2 College of Chemistry and Chemical Engineering, Xi’an Shiyou University, Xi’an, 710065 Shaanxi China; 3 Shaanxi Province Key Laboratory of Environmental Pollution Control and Reservoir Protection Technology of Oilfields, Xi’an, 710065 China; 4 State Key Laboratory of Petroleum Pollution Control, CNPC Research Institute of Safety and Environmental Technology, Beijing, 102206 China

**Keywords:** Melamine, Cross-linked polycation, Inhibitor, Clay, Swelling

## Abstract

In this paper, a series of poly ammonium shale inhibitors were prepared from diethylamine, epichlorohydrin, and melamine (DEM) and their inhibition to shale were evaluated by bentonite linear expansion test, anti-swelling experiments, and mud ball experiments. Additionally, other properties of drilling fluid treated by poly ammonium were evaluated. Anti-swelling results showed that anti-swelling rate of DEM-8 reaches up to 97.8% when its concentration reaches to 0.8%. Mud ball experiment and drilling fluid evaluation showed DEM-8 has strong inhibitive capability to bentonite hydration swelling and controlling the particle size of bentonite in a large scale. The inhibition mechanism of DEM-8 was studied by thermogravimetric analysis and scanning electron microscope. The results demonstrate that DEM-8 can be adsorbed on clay surface through electrostatic interaction and hydrogen bonds by an anchoring effect and a hydrophobic effect.

## Introduction

Shale oil/gas has been one of the technologies highlighted in the world in recent years. During the drilling, borehole stability problems such as bit balling, disintegration of cuttings, borehole wash-out and stuck pipe mostly occur in shale formations due to hydration and swelling of water-sensitive shales [1–3]. When water-sensitive shales (high montmorillonite content) are exposed to water-based drilling fluids, depending on the chemical characteristics of the shale or drilling fluid, this can result in a rapid swelling or dispersion of the shale [4]. Consequently, a high level of shale inhibitor has been utilized widely in drilling operations [4, 5], but same additives may be unfavorable due to the environmental protection requirements, which limits their usage or restricts their discharge [6]. Recently, organic amine compounds with high performance as shale inhibitor have drawn much attention of the researchers. This kind of inhibitor has obtained wide application around the world with great success because of its excellent inhibition, lubrication and stable rheological property and so on [7, 8]. As the polyamine salt has higher inhibitory and anti-balling abilities, and it is not poisonous and hazardous, the use of this drilling fluid could decrease the cost of oil contaminated drilled cuttings disposal [9, 10]. Currently, polyamine compound can be used in various kinds of water-based oilfield working fluid and has superior compatibility with traditional additives, and it can meet environmental protection requirements, due to its oxidation product is harmful for animals [11]. In the current work, the inhibitive properties of a melamine cross-linking agent are evaluated through experiments including linear expansion, mud balls, particle distribution measurements, thermogravimetric analysis and scanning electron microscopy. Furthermore, the inhibitive mechanism is discussed in detail.

In this paper, a new shale inhibitor with high stability has been synthesized from diethylamine, epichlorohydrin, and melamine (DEM) and their inhibitions to shale have been evaluated in detail. Both the effect of the polymer to the properties of drilling fluid and the proposed inhibition mechanism have also been disused.

## Materials and methods

### Materials

The drilling fluid were constructed using several additives, diethylamine and pichlorohydrin were provided by Sinopharm Chemical Reagent Co. Melamine was purchased from Tianjin Kemiou Chemical Reagent Co. Ltd., China. Modified Xanthan Gum and modified starch were all supplied in domestic market. Bentonite was obtained from Changqing Bentonite Group Co., Ltd., China.

### Synthesis of DEM

**Scheme 1:**
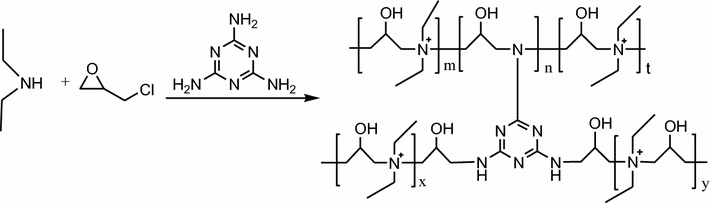
Synthesis of cross-linked poly-ammonium (DEM)

Diethylamine and epichlorohydrin with the mole ratio of 1:1 as well as melamine used as the cross-linking agent were employed to synthesize shale inhibitor under 60 °C [12, 13], as shown in Scheme 1, and the final product, melamine cross-linking agent, was abbreviated as DEM in the following text.

### Swelling inhibition and mud ball immersing test

The hydration swelling of bentonite is tested by a NP-01 shale expansion instrument (Haitongda, Co., Ltd., Qingdao, China), in accordance with Chinese petroleum and natural gas industry standards SY/T6335-1997 and SY/T5971-1994. Mud ball immersing test is as follows: bentonite (10 g) was used to make a mud ball, and the mud ball was immersed in 80 mL tap water or other aqueous solutions for 24 h [14, 15]. Then the details of the immersed mud balls were evaluated, including a check whether there are cracks or dilapidation on the surface.

### Drilling fluid properties evaluation experiment

4% (m/m) bentonite was dispersed in 350 mL of water containing certain amount of inhibitor [16]. After stirring for 20 min, aged for 16 h at room or high temperature, then the rheological properties and filtration of the fluid samples were measured using a model ZNN-D6S viscometer (Haitongda, Co., Ltd., Qingdao, China), including Apparent viscosity (AV), Plastic viscosity (PV), Yield point (YP), Dynamic plastic ratio (YP/PV), API Filtration (FL) and Friction coefficient (tg) [17]. The apparent viscosity, plastic viscosity and yield point were calculated from 300 and 600 rpm readings using following formulas from petroleum and natural gas industry standards for field testing of drilling fluids (GB/T 16783.1-2006):

$$ \begin{aligned} {\text{Apparent viscosity }}\left( {\text{AV}} \right) \, = \,\upphi_{ 600} / 2 { }\left( {\text{mPa s}} \right) \hfill \\ {\text{Plastic viscosity }}\left( {\text{PV}} \right) \, = \,\upphi_{ 600} -\upphi_{ 300} \left( {\text{mPa s}} \right) \hfill \\ {\text{Yield point }}\left( {\text{YP}} \right) \, = \,\upphi_{ 600} - {\text{AV }}\left( {{\text{N}}/{\text{m}}^{ 2} } \right). \hfill \\ \end{aligned} $$

### Particle distribution test

4% (m/m) bentonite dispersion was prepared and prehydrated for 24 h. Inhibitors with certain concentrations were added into the dispersion and stirred for 20 min, after aged for 24 h, and then the size distribution of the particles was measured by LS-13320 laser particle size analyzer based on the light scattering principle (Beckman Coulter, Inc., USA) using equipment operating procedure under the pump speed of 40%.

### TGA and SEM

After the bentonite was dispersed in inhibitor solutions for 24 h, the bentonite was separated and dried at 105 °C for thermogravimetric analysis (TGA) and scanning electron microscopy (SEM). TGA experiment was performed on a TGA/DSC 1/1600 thermal analysis machine (METTLER TOLEDO, Inc., Switzerland) at a ramp of 20 °C/min from room temperature to 825 °C under nitrogen flow. The surface morphology of the sample under study in the absence and presence of inhibitors was investigating using a Digital Microscope Imaging scanning electron microscope (model SU6600, serial No. HI-2102-0003) at accelerating voltage of 40.0 kV. Samples were attached on the top of an aluminum stopper by means of carbon conductive adhesive tape. All micrographs of the specimen were taken at 5009 times magnification.

## Results and discussion

### Selection of the concentration of cross-linking agent and synthesis temperature

The influence of the amount of cross-linking agent and synthesis temperature on the performance of the inhibitor was investigated by the clay-swelling rate, and the results were shown in Table 1. Obviously, when the concentration of cross-linking agent is 0.1%, the anti-swelling rate of clay is the lowest. So it is advisable to choose 0.1% cross-linking agent in the following experiment. Furthermore, under different temperature, 0.1% cross-linking agent was investigated on the effect of clay-swelling rate. The results show that the product synthesized under 90 °C, DEM-8, displays most potent inhibition with the lowest clay-swelling rate of 53.75% within 90 min. The possible reason may attribute to the proper ratio of cross-linking agent helping to form a cross-linked net work, and the low ratio and low temperature is not efficient to cross link the polyammonium while the high ratio and high temperature may lead the polyammonium to cross link and assemble into a tight agglomeration. So DEM-8 was selected for the further study in detail.

**Table 1:** The conditions of synthesis polycation inhibitors

Name	Cross-linking dosage/%	T (/°C)	Swelling rate, /% (90 min)
DEM-1	0.02	120	63.18
DEM-2	0.05	120	59.15
DEM-3	0.1	120	57.79
DEM-4	0.3	120	60.21
DEM-5	0.5	120	62.45
DEM-6	0.1	25	55.80
DEM-7	0.1	60	55.12
DEM-8	0.1	90	53.75
DEM-9	0.1	150	64.50

### Swelling inhibition

**Fig. 1:**
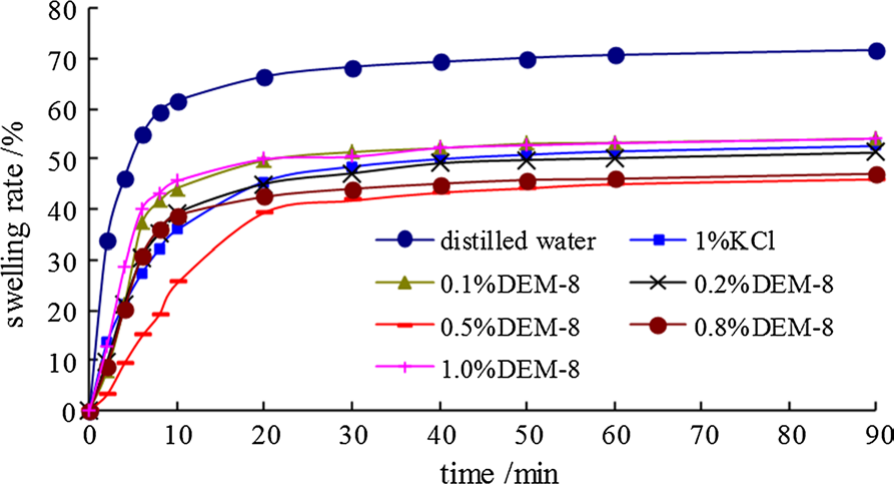
The screening of the concentration of inhibitors

In order to investigate the influence of inhibitor to the swelling inhibition of bentonite, the swelling rate with time in different concentration of inhibitor solutions was recorded. As shown in Fig. 1, independent of net-work poly-quaternary ammonium salts addition, the swelling rate increases dramatically during the first 10 min, followed by a slow increase. Compared with the blank sample, inhibitor solution shows stronger inhibition to hydration and swelling of bentonite. The swelling rate reaches to a minimum at a certain concentration in DEM-8 solution. The inhibition depends on the adsorption of poly-quaternary ammonium salts on the clay surface through electrostatic interaction and hydrogen bonds by an anchoring effect and a hydrophobic effect. Since the inhibition has complex function, the swelling rate does not show a linear relation with the concentration. According to results from comprehensive testing, 0.5% DEM-8 may be an optimum.

**Fig. 2:**
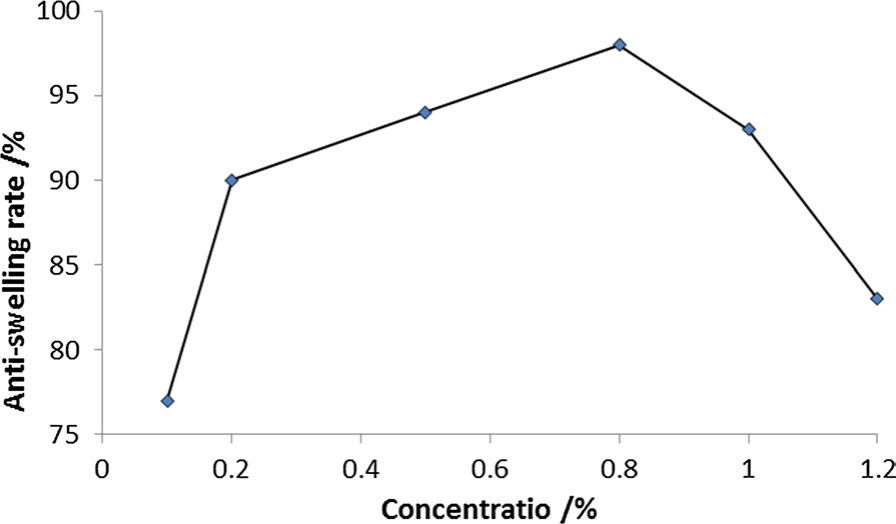
The effect of DEM-8 concentration on anti-swelling rate

A centrifugal separation technique was used to determinate the influence of DEM to the anti-swelling property of clay. This was used in accordance with petroleum and natural gas industry standard SY/T5971-1994 of China. The results show that anti-swelling rate of DEM presents a tendency of increase first and then reduce with the increase of its concentration. Obviously, anti-swelling rate of 0.8% DEM-8 reaches up to the highest, 97.8% (Fig. 2).

**Fig. 3:**
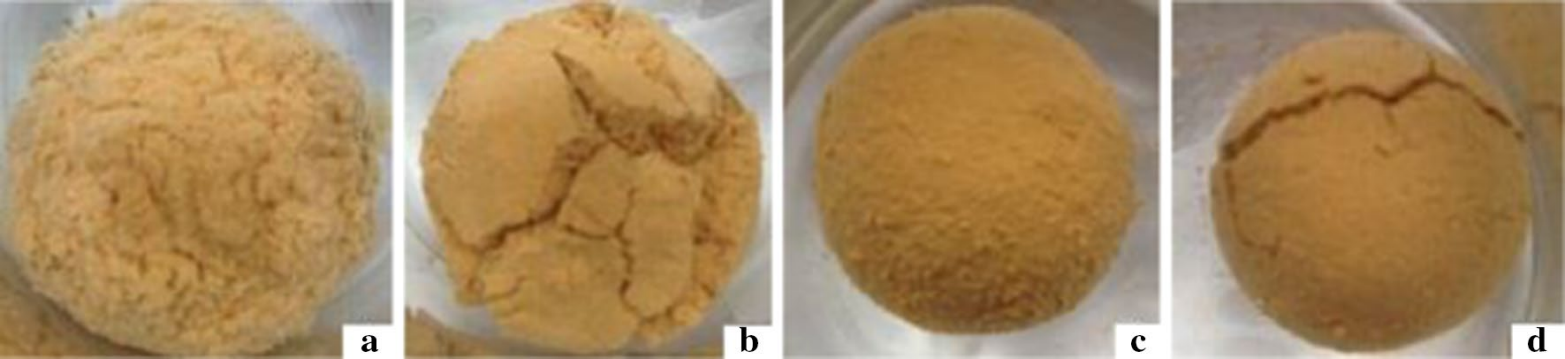
The appearance of mud balls immersed in different treatment solutions for 12 h. **a** Distilled water, **b** 0.8% DEM-8, **c** 1.0% KCl, **d** 0.8% DEM-8 + 1.0% KCl

The mud ball immersing test provides a more intuitive way to describe the inhibition property of DFP-8. The mud balls were immersed into water and 0.8% DEM-8 and 1.0% KCl respectively. In Fig. 3, it is shown the status of the mud balls after immersed for 24 h. The mud ball immersed in water swells obviously, and the surface is loose. The mud ball immersed in 1.0% KCl swells not clearly, and the surface is smooth and free of cracks, while the mud ball immersed in 0.8% DEM-8 solution swells slightly, and the surface is smoother and cracked. It is clear that DEM has a significantly strong clay-swelling inhibitory capability. This phenomenon could be explained by a surface film on the clay resulting from absorption of DEM, which blocks the water penetration into the clay and prevent clay from hydrating swelling.

### Effect of rheological property and filtration of drilling fluids

The effects of DEM on the rheological performance of drilling fluids measured in accordance with GB/T 16783.1-2006 [18] were shown in Table 2. As can be seen from Table 2, with adding of DEM-8, the viscosity and shearing force of the drilling fluid lower than the control sample without inhibitor, but the filtration property does not appear significant change, indicating that DEM-8 can effectively restrain the bentonite slurry to reduce the viscosity. The synthesized DEM-8 and traditional drilling fluid additives have excellent de-filtration effect. The DEM-8 is compatible with the conventional additives in water-based drilling fluids. What’s the most important thing is that DEM-8 can effectively improve the performance of polysaccharide at higher temperature, at least under 120 °C.

**Table 2:** Evaluation results of drilling fluid rheological properties

T (/°C)	Additives	AV (/mPa s)	PV (/mPa s)	YP (/Pa)	YP/PV (Pa/mPa s)	FL (/mL)	tg
25	Blank	3.2	1.9	1.2	0.6221	11.8	0.0628
	0.01% DEM-8	2.5	1.5	0.8	0.4892	14.9	0.0782
	2.0% KD-03	6.9	2.7	3.7	1.2436	6.3	0.1056
	2.0% modified xanthan gum	7.4	4.4	3.0	0.6213	5.4	0.2019
	2.0% KD-03 + 0.01% DEM-8	4.8	3.3	1.5	0.4126	8.0	0.0716
	2.0% starch + 0.01% DEM-8	5.0	3.0	1.9	0.5987	5.8	0.0473
120	Blank	2.4	1.7	0.6	0.3616	13.6	0.1698
	0.01% DEM-8	1.9	1.3	0.6	0.4659	18.5	0.1127
	2.0% KD-03	5.4	4.8	0.4	0.0784	10.5	0.1314
	2.0% modified xanthan gum	10.1	8.7	2.0	0.2347	6.3	0.1221
	2.0% KD-03 + 0.01% DEM-8	4.1	2.9	1.1	0.3521	10.1	0.0716
	2.0% starch + 0.01% DEM-8	8.4	6.5	1.7	0.2498	5.0	0.0472
150	Blank	2.8	2.6	0.1	0.0318	14.8	0.0541
	0.01% DEM-8	2.1	1.5	0.5	0.3011	16.3	0.0978
	2.0% KD-03	4.8	3.1	1.6	0.4821	15.1	0.0391
	2.0% modified xanthan gum	6.2	3.7	2.5	0.5987	10.1	0.0911
	2.0% KD-03 + 0.01% DEM-8	3.2	2.0	1.2	0.5418	16.1	0.0619
	2.0% starch + 0.01% DEM-8	6.7	4.0	2.6	0.5986	10.1	0.0554

### Inhibitive mechanism analysis

#### Particle size distribution test

**Fig. 4:**
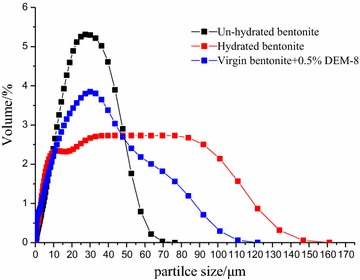
The distribution of clay particle size in different suspensions

Figure 4 shows the particle size distribution of the bentonite dispersions when these are treated in different ways. Compared to the un-hydrated control sample, the average size of hydrated bentonite particles increased from 16.25 to 32.94 μm. This is different when DEM was added before the hydration of bentonite. When DEP was added before the virgin bentonite was dispersed into water, after 16 h, the average size of clays changed slightly, and it is just a litter larger than that of virgin bentonite, from 16.25 to 25.00 μm, which indicates that DEM-8 can inhibit the bentonite swelling effectively.

#### Thermogravimetric analysis

**Fig. 5:**
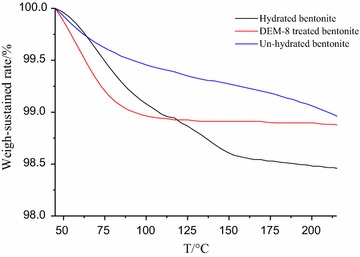
The TGA of the bentonite treated with different methods

Thermogravimetric analysis (TGA) measure a mass loss of the hybrids as a function of temperature. The decomposition of organic–inorganic hybrids would take place in four steps: water desorption, dehydration, decomposition of polyammonium and dehydroxylation [19]. As shown in Fig. 5, several mass loss steps are observed in the curves of bentonite immersed in tap water and 0.8% DEM-8 solution. The thermal degradation of the DEP treated bentonites differs significantly from that of the control bentonite between room temperature and 215 °C. The weight loss of bentonite treated with tap water is 1.5% from 45 to 200 °C, while the weight loss of 0.5% DEM-8 modified bentonite is 1.2%. From this test, it can be concluded that 0.8% DEM-8 can inhibit the water absorption of bentonite.

#### Scanning electron microscopy

**Fig. 6:**
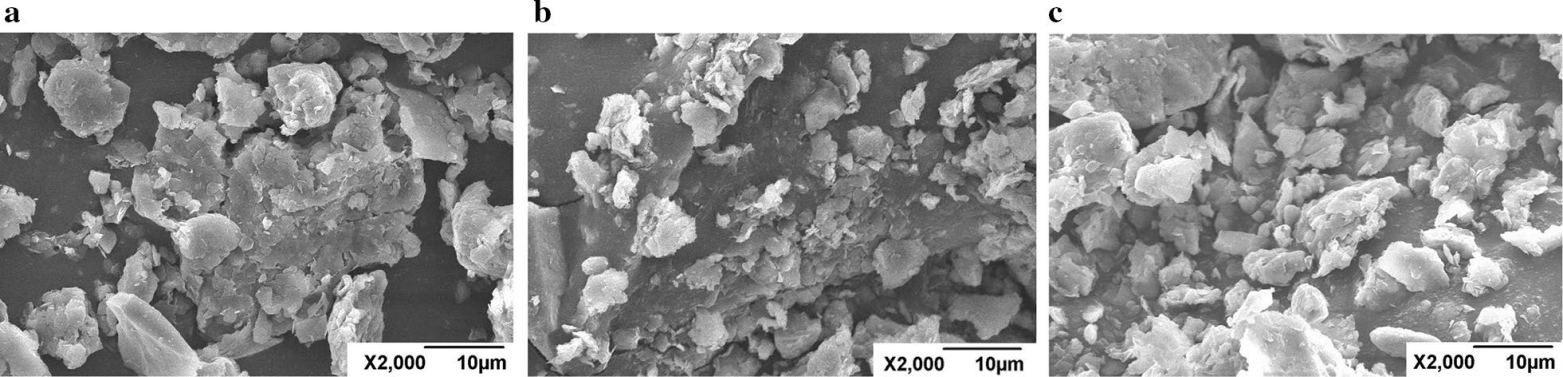
SEM pictures of bentonite treated with different methods. **a** Virgin bentonite, **b** bentonite treated with water, **c** bentonite treated with DEM-8

In order to evaluate the morphology of bentonite particles treated by different ways, scanning electron microscopy (SEM) was carried out. Figure 6a shows an SEM image of the virgin bentonite without any treatment. Figure 6b shows the bentonite after immersed in water for 24 h, and Fig. 6c shows SEM images of the bentonite after immersed in DEM-8 for 24 h. From the three pictures, it is observed that after immersed in water, the particles dispersed and change to smaller particles. However, the particle size of 0.5% DEM-8 treated sample is similar than that of the virgin sample but much larger than that of tap water treated sample. It is anticipated that, except for electrostatic interaction, the hydrogen bonding between amium group, hydroxyl group and siloxane of clay and modification of surface affinity toward water can further restrict the swelling and hydration of clay minerals [20].

## Conclusions

In this work, cross-linked polycation inhibitor (DEM) was synthesized with epichlorohydrin, diethylamine and melamine. The inhibitive properties of DEM-8 to clays swelling were investigated linear expansion test, mud ball immersing test etc. The results indicate that, compared with a blank solution, DEM-8 shows stronger inhibition to hydration and swelling of bentonite. The anti-swelling rate of 0.5% DEM-8 reaches up to 85%. The hydration expansion degree of the mud ball in the DEM-8 solution was significantly weaker than that of blank. The inhibition mechanism of DEM-8 to shales may be due to the ion exchange, hydrogen bonding, anchoring effect and modification of surface affinity toward water. This was found by thermogravimetric analysis, ion exchange test and scanning electron microscope analysis.
